# Nrf2-Mediated HO-1 Induction Coupled with the ERK Signaling Pathway Contributes to Indirect Antioxidant Capacity of Caffeic Acid Phenethyl Ester in HepG2 Cells

**DOI:** 10.3390/ijms150712149

**Published:** 2014-07-09

**Authors:** Jin-Kyoung Kim, Hae-Dong Jang

**Affiliations:** Department of Food and Nutrition, Hannam University, Daejeon 305-811, Korea; E-Mail: kikkne@naver.com

**Keywords:** caffeic acid phenethyl ester (CAPE), indirect antioxidant activity, heme oxygenase 1 (HO-1), nuclear transcription factor-erythroid 2-related factor 2 (Nrf2) accumulation, ERK (extracellular signal-regulated kinase) phosphorylation

## Abstract

The objective of this study is to investigate the contributing effect of the nuclear transcription factor-erythroid 2-related factor 2 (Nrf2)-mediated signaling pathway on the indirect antioxidant capacity of caffeic acid phenethyl ester (CAPE) against oxidative stress in HepG2 cells. The result of an antioxidant response element (ARE)-luciferase assay showed that CAPE stimulated ARE promoter activity resulting in increased transcriptional and translational activities of heme oxygenase-1 (HO-1). In addition, CAPE treatment enhanced Nrf2 accumulation in the nucleus and the post-translational phosphorylation level of extracellular signal-regulated kinase (ERK) among several protein kinases tested. Treatment with ERK inhibitor U126 completely suppressed CAPE-induced ERK phosphorylation and HO-1 expression, but it only partly inhibited CAPE-induced Nrf2 accumulation and ARE promoter. Using the 2',7'-dichlorofluorescein-diacetate (DCFH-DA) method, the cellular antioxidant capacity of CAPE against 2,2'-azobis (2-amidinopropane) dihydrochloride (AAPH)- or H_2_O_2_-induced oxidative stress also was shown to be partially suppressed by the ERK inhibitor. From the overall results it is proposed that the indirect antioxidant activity of CAPE against oxidative stress in HepG2 cells is partially attributed to induction of HO-1, which is regulated by Kelch-like erythroid-cell-derived protein with CNC homology (ECH)-associated protein 1 (Keap1)-independent Nrf2 activation relying on post-translational phosphorylation of ERK.

## 1. Introduction

The cellular protection against oxidative stress can be carried out by antioxidants in direct and indirect ways depending upon the type of working mechanism [1]. Direct antioxidants, which are always redox active, scavenge reactive oxygen and nitrogen radical species by being consumed or chemically modified. They have to be replenished or regenerated. In contrast with direct antioxidants, indirect antioxidants may or may not be redox active. Indirect antioxidants exert their antioxidant effects through upregulating phase II detoxifying and antioxidant enzymes [1]. Some antioxidants such as phenolic Michael reaction acceptors may display their antioxidant effects in both a direct and an indirect fashion [2] and are called bifunctional antioxidants. 

The activation of nuclear transcription factor-erythroid 2-related factor 2 (Nrf2), including the release of Nrf2 from Kelch-like ECH-associated protein 1 (Keap1)-Nrf2 complex, is involved in the induction of gene encoding detoxifying and antioxidant enzymes by indirect antioxidants including oxidizable diphenol [3]. Nrf2 may be activated in a Keap1-dependent or -independent manner [4,5]. For Keap1-dependent NRf2 activation, a conformational change of Keap1 through interaction with different types of inducers is required [6]. In Keap1-independent Nrf2 activation, Nrf2 protein may be phosphorylated by several signal transduction pathways including mitogen-activated protein kinase (MAPK), phosphatidylinositol 3-kinase (PI3K/Akt), protein kinase C (PKC), extracellular signal-regulated kinase (ERK), and c-jun *N*-terminal kinase (JNK) signaling pathways [7,8]. 

Caffeic acid phenethyl ester (CAPE) is a phenolic compound bearing one catechol group and is one of the biologically active constituents present in propolis from honeybee hives. CAPE has been reported to have biological and pharmacological activities including antioxidant [9,10,11,12,13,14], anti-inflammatory [15,16], antiviral [17], anti-carcinogenic [18], and immunomodulating effects [6]. CAPE has demonstrated a potent antioxidant activity in previous studies using an* in vitro* model [9,10,11,12] as well as an* in vivo* animal model [13,14]. The strong free radical-scavenging and ferric reducing activity of CAPE has been reported [9,11]. Also the potential cellular antioxidant activity of CAPE against oxidative stress induced by menadione has been confirmed in human umbilical vein endothelial cells [12], while CAPE has shown a protective effect on lipid peroxidation and antioxidant enzymes in diabetic rat organs such as the liver and heart [13,14].

Although CAPE has been shown to stimulate heme oxygenase-1 (HO-1) through promoting inactivation of the Nrf2-Keap1 complex in renal epithelial cells [19], there has been no study to elucidate the induction of phase II detoxifying and antioxidant enzymes through Nrf2 activation in hepatic cells. Human hepatoma HepG2 cell, a well-differentiated transformed cell line, is used in this study. Because steady-state functioning of the antioxidant defense system in HepG2 cells is relatively higher than that in hepatocytes and other non-transformed cells, the variations of cellular responses to different treatments can be more conveniently measured [20,21]. The purpose of our present study is to investigate the contribution of Nrf2-mediated HO-1 expression to CAPE protection against oxidative stress in hepatic HepG2 cells. We propose that CAPE exerts indirect antioxidant capacity by up-regulating the expression of phase II antioxidant and detoxifying enzyme HO-1 via the ERK-Nrf2 signaling pathway.

## 2. Results and Discussion

### 2.1. CAPE (Caffeic Acid Phenethyl Ester) Increases ARE (Antioxidant Response Element)-Luciferase Activity and HO-1 (Heme Oxygenase-1) Expression

**Figure 1:**
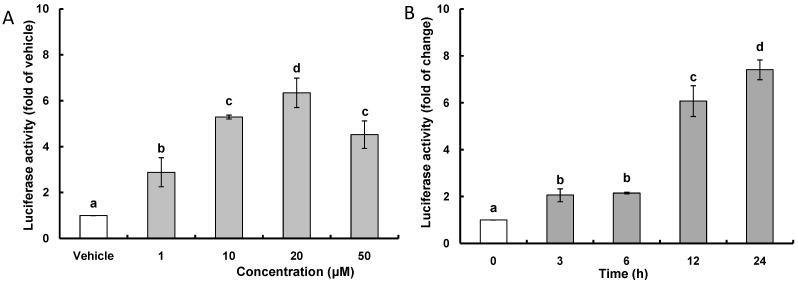
CAPE (caffeic acid phenethyl ester) increases ARE (antioxidant response element) luciferase activity. (**A**) After HepG2 cells were subjected to 1–50 μM CAPE for 12 h, ARE luciferase activity was determined and normalized to vehicle; (**B**) Time-course experiment was carried out for the indicated times using 20 μM CAPE and normalized to time zero. Data are expressed as mean ± standard deviation of three individual experiments. Different corresponding letters indicate significant differences at *p* < 0.05 by Duncan’s test.

**Figure 2:**
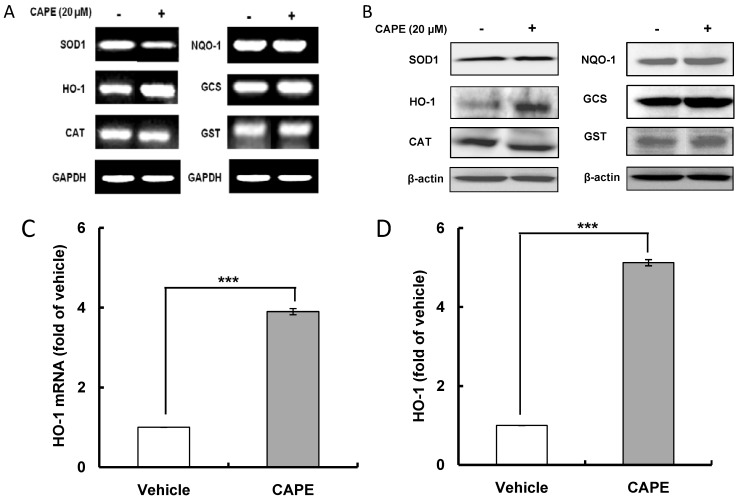
CAPE enhances HO-1 (heme oxygenase-1) mRNA and protein levels. After HepG2 cells were subjected to 20 μM CAPE for 12 h, the transcription and translation levels of phase II antioxidant and detoxifying enzymes in HepG2 cells were determined using reverse transcription-polymerase chain reaction (RT-PCR) and western blot analysis respectively. (**A**) mRNA level of phase II antioxidant and detoxifying enzymes; (**B**) Protein level of phase II antioxidant and detoxifying enzymes; (**C**) CAPE increases HO-1 mRNA level; (**D**) CAPE augments HO-1 protein level. Data are expressed as mean ± standard deviation of three individual experiments, *** *p* <0.001* vs.* vehicle. SOD1, superoxide dismutase 1; HO-1, heme oxygenase-1; CAT, catalase; NQO1, NAD(P)H:quinone oxidoreductase 1; GCS, glutamylsysteine synthase; GST, glutathione S-transferase.

ARE is a *cis*-acting DNA regulatory element present in the promoter/enhancer regions of genes encoding many antioxidant and detoxifying enzymes; and to test whether or not CAPE, as an indirect antioxidant, stimulates the transcription of ARE-related gene in HepG2 cells, a luciferase reporter plasmid carrying ARE promoter was introduced to HepG2 cells. The dose-response effects of CAPE on ARE prompter activity were examined over 24 h, and the results of an ARE-luciferase assay showed that CAPE treatment caused a concentration-dependent induction of ARE promoter activity with peak activity seen at 20 μM (Figure 1A); therefore, 20 μM was used as the concentration of reference in all subsequent experiments. As a time-course study showed that there was a sufficient increase in ARE promoter activity after 12 h with a 6.1 fold change compared with a 7.4 fold change after 24 h (Figure 1B), the effects of CAPE on transcriptional and translational activity of phase II antioxidant and detoxifying enzymes was examined 12 h after treatment with CAPE. Results for the transcription level of phase II antioxidant and detoxifying enzymes in treatment with 20 μM CAPE for 12 h (Figure 2A,C) and their translation level (Figure 2B,D) show that significant increases in both transcription and translation activity were observed only in HO-1 among the phase II antioxidant and detoxifying enzymes and were 3.9 and 5.1 fold of vehicle respectively. These data indicate that the increase in ARE activity observed with CAPE treatment is likely to be closely related with an increase of HO-1 transcriptional activity. This inducing effect of antioxidant-related gene by CAPE has been confirmed through studies with human umbilical vein endothelial cells that showed that CAPE suppressed menadione-induced oxidative stress by inducing HO-1 gene [22,23] and with porcine renal epithelial cells showed that CAPE significantly increased HO-1 protein expression through stimulating Nrf2 expression [19]. The inducing effect of CAPE on phase II antioxidant and detoxifying enzymes such as HO-1 and superoxide dismutase (SOD) has also been identified in different types of cells such as astrocytes [24], hippocampal neurons [25], macrophage cells [26], and 3T3-L1 adipocytes [27]. Accordingly, the above results suggest that CAPE can exert potent intracellular antioxidant activity by inducing HO-1 expression.

### 2.2. CAPE Activates ERK (Extracellular Signal-Regulated Kinase) Leading to Nrf2 (Nuclear Transcription Factor-Erythroid 2-Related Factor 2) Accumulation in the Nucleus

**Figure 3:**
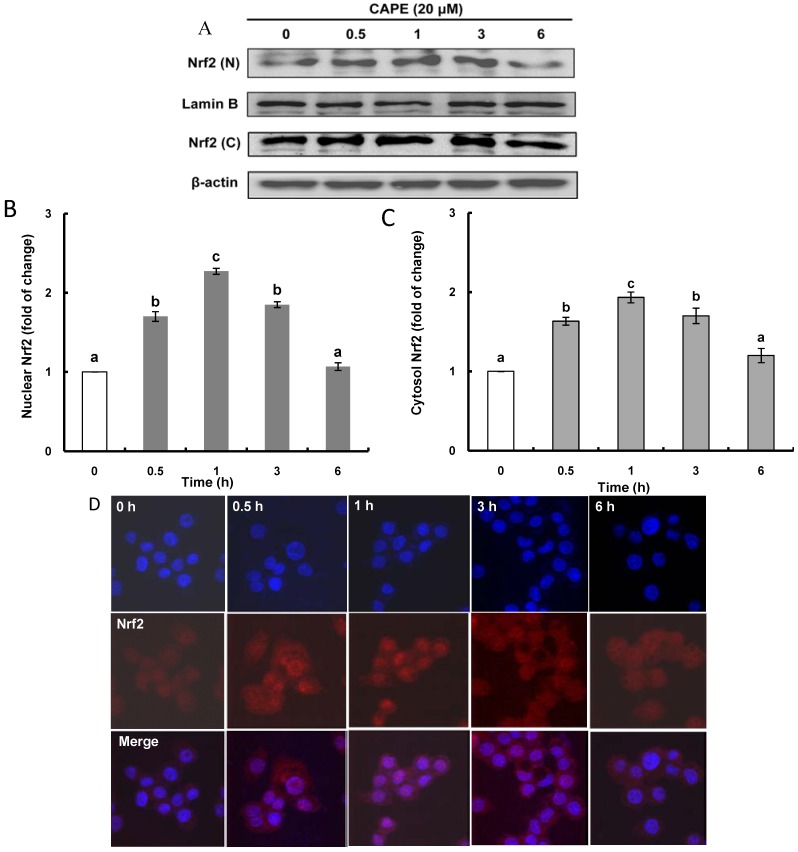
CAPE increases Nrf2 (nuclear transcription factor-erythroid 2-related factor 2) level in the nucleus and the cytosol. (**A**) After HepG2 cells were incubated with 20 μM CAPE for 6 h, the time-course induction of Nrf2 protein levels in the nucleus and the cytosol was determined using western blot analysis and normalized to time zero; (**B**) Time-course change of Nrf2 protein level in the nucleus; (**C**) Time-course change of Nrf2 protein level in the cytosol; and (**D**) Immunofluorescence staining of HepG2 cells subjected to 20 μM CAPE as indicated with anti-Nrf2 antibodies. Nuclear counterstaining was done with Hoechst 33528 (Sigma Aldrich, St. Louis, MO, USA). Fluorescence image was taken by confocal fluorescence microscopy. Data are expressed as mean ± standard deviation of three individual experiments. Different corresponding letters indicate significant differences at *p* < 0.05 by Duncan’s test.

It is known that nuclear factor Nrf2 is the major transcription factor that binds to ARE and it exists as Keap1/Nrf2 complex in the cytosol. For the induction of phase II antioxidant and detoxifying enzymes, Nrf2 has to be released as an activated form from the Keap1-Nrf2 complex by Keap1-dependent or -independent mechanisms and to be translocated from the cytosol into the nucleus. To examine Nrf2 induction by CAPE, the protein levels of Nrf2 in the nucleus and the cytosol were analyzed, and a time-course study revealed that CAPE caused time-dependent increases of Nrf2 translation in both the nucleus and the cytosol with a peak level 1 h after treatment with CAPE (Figure 3A–C). These elevated levels of Nrf2 protein in both the nucleus and the cytosol by CAPE treatment were also observed by fluorescence imaging with confocal microscopic analysis (Figure 3D). The enhanced Nrf2 level in the cytosol with CAPE treatment may be due to autoregulation, the ability of Nrf2 to regulate its own transcription. As ARE elements recruiting Nrf2 are found in close proximity of the promoters of Nrf2-related antioxidant genes as well as Nrf2 itself [4], CAPE may induce Nrf2 transcription in cells, resulting in an increase of Nrf2 protein level in the cytosol. The increased Nrf2 protein level in the nucleus with CAPE treatment could be explained by two plausible mechanisms of Nrf2 activation, Keap1-dependent or Keap1-independent. Keap1-dependent Nrf2 activation requires strong electrophiles disrupting the Keap1-Nrf2 complex by modifying Keap1 at cysteine residues through the Michael reaction [4]. Meanwhile, post-translational phosphorylation through multiple kinase signaling pathways is known to be involved in Keap1-independent Nrf2 activation. 

The induction of phase II antioxidant and detoxifying enzymes through Nrf2 activation by CAPE was reported in previous studies using different cells such as renal epithelial cells and lung fibroblasts [19,28]. From these reports, it is postulated that CAPE phosphorylates Nrf2 causing it to be released from the Keap1/Nrf2 complex and to be translocated from the cytosol into the nucleus, which suggests that CAPE may activate Nrf2 in a Keap1-independent manner. It has been proposed that there may exist several Keap1-independent mechanisms of Nrf2 activation including phosphorylation of Nrf2 by various protein kinases, interaction with other proteins, and epigenic factors [4]; and a number of protein kinases such as MAPK, ERK, JNK, p38 Akt, and PKC have been implicated as an upstream signal in the regulation of Nrf2 activity [7]. 

**Figure 4:**
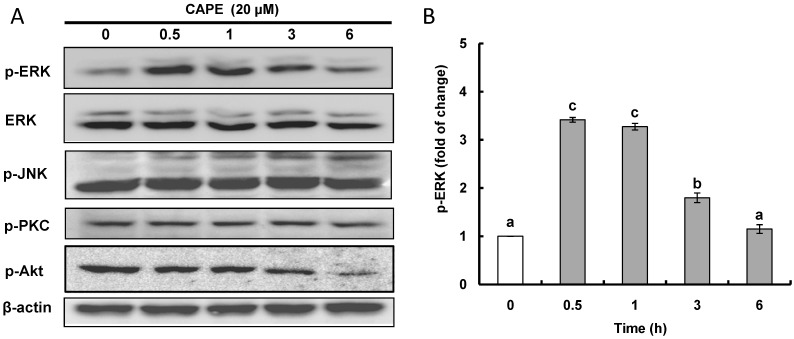
CAPE activates ERK (extracellular signal-regulated kinase) protein kinase. (**A**) After HepG2 cells were subjected to 20 μM CAPE for the indicated times, the phosphorylated protein kinases in HepG2 cells was determined using western blot analysis; (**B**) Time-course induction of ERK protein kinase normalized to time zero. Data are expressed as mean ± standard deviation of three individual experiments. Different corresponding letters indicate significant differences at *p* < 0.05 by Duncan’s test.

To investigate whether any protein kinases such as ERK, JNK, PKC, and Akt may be involved in the activation (post-translational phosphorylation) of Nrf2, the phosphorylated level of different protein kinases with CAPE treatment was analyzed (Figure 4A,B). It was found that CAPE treatment only had a notable inducing effect on ERK phosphorylation. A time-course experiment showed that phosphorylated ERK increased with a peak level (3.4 fold change) at 0.5 h after CAPE treatment (Figure 4B). This observation supports the proposal that CAPE may activate ERK by inducing its phosphorylation and in turn phosphorylate Nrf2, known as a downstream signal, which results in Nrf2 being activated and translocated to the nucleus. Nrf2 activation in hepatoma HepG2 cells through ERK protein kinase was also observed with icariside and butylated hydroxyanisole (BHA) [29,30]. Icariside enhanced Nrf2 activation leading to HO-1 and GST expression through the ERK as well as Akt and JNK signaling pathways, while BHA also increased Nrf2 activation causing HO-1 expression through the ERK as well as JNK signaling pathways.

### 2.3. CAPE Induces HO-1 Expression through the ERK-Nrf2 Signaling Pathway

**Figure 5:**
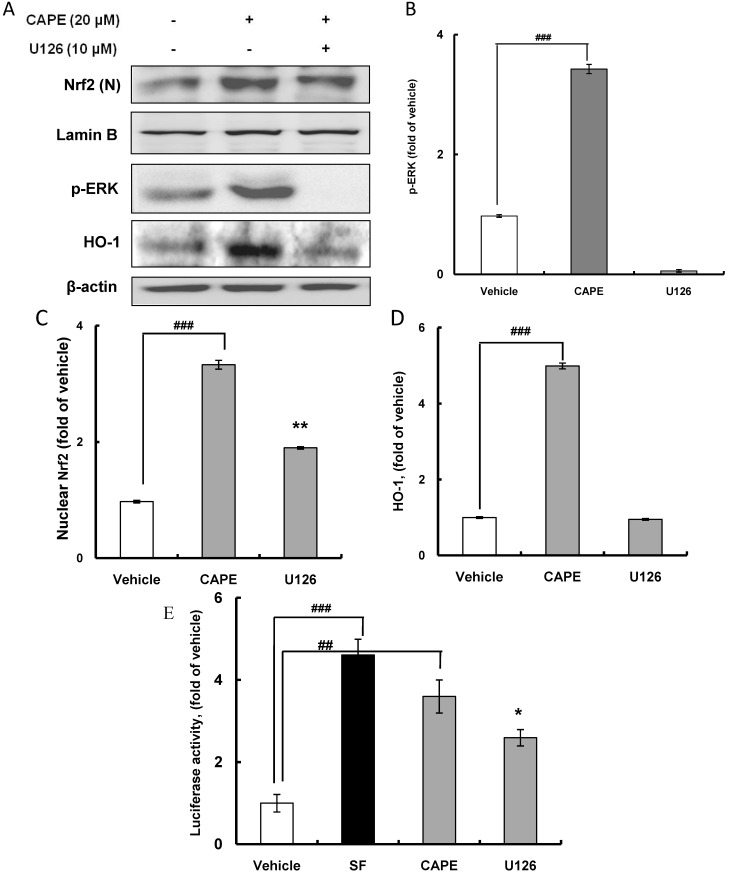
HO-1 induction requires Nrf2 accumulation in the nucleus via HO-1 activation. (**A**) After HepG2 cells were subjected to 20 μM CAPE for 30 min in the presence or absence of U126, ERK inhibitor, Nrf2 in the nucleus, phosphorylated ERK, and HO-1 were determined using western blot analysis; (**B**) U126, ERK inhibitor prevents the activation of ERK; (**C**) U126, ERK inhibitor, suppresses Nrf2 accumulation in the nucleus; (**D**) U126, ERK inhibitor, prevents HO-1 expression; and (**E**) U126, ERK inhibitor, suppresses ARE promoter activity normalized to vehicle. Sulforaphane (SF) was used as a positive control inducing ARE promoter activity. Data are expressed as mean ± standard deviation of three individual experiments, ^##^ *p* <0.01, ^###^ *p* <0.001* vs.* vehicle, * p <0.05, ** p < 0.01* vs.* CAPE.

Nrf2-dependent HO-1 expression coupled with the ERK signaling pathway was examined by western blot analysis using ERK inhibitor U126. As expected, treatment with U126 potently abolished ERK activation (Figure 5A,B). Furthermore, CAPE treatment significantly (*p* < 0.001) augmented nuclear accumulation of Nrf2 by 3.3 fold, and this was clearly ameliorated to 1.9 fold by treatment with U126 (Figure 5C), suggesting that ERK phosphorylation by CAPE may activate Nrf2 by post-translational phosphorylation and allow translocation of Nrf2 to the nucleus. In addition, U126 treatment obviously diminished HO-1 expression, which was significantly (*p* < 0.001) promoted 5.0 fold by CAPE treatment in comparison with the vehicle level (Figure 5D). Moreover, treatment with U126 significantly (*p* < 0.05) reduced ARE promoter activity which was also significantly (*p* < 0.01) stimulated by CAPE (Figure 5E). In this experiment, sulforaphane (SF) was used as a positive control of inducer of ARE promoter activity. Accordingly, CAPE phosphorylates ERK to induce the post-translational Nrf2 phosphorylation, which is required for Nrf2 accumulation in the nucleus leading to HO-1 expression. Thus, this Nrf2 activation coupled with the ERK signaling pathway for inducing HO-1 expression appears to be Keap1-independent. Taken together, overall results imply that CAPE may activate Nrf2 through activating ERK to contribute to the induction of phase II antioxidant and detoxifying enzymes including HO-1 in hepatic HepG2 cells. 

On the other hand, the blocking of ERK activation by U126 did not completely suppress Nrf2 accumulation in the nucleus, indicating that the Keap1-dependent pathway may be involved in Nrf2 activation for translocation to the nucleus as well as the Keap1-independent signaling pathway through ERK phosphorylation. In a previous study using human colon carcinoma HCT116 cells, oxidized CAPE showed greater potency than CAPE in activating the Nrf2 pathway, which was confirmed by increased induction of HO-1, ARE promoter activity, and nuclear accumulation of Nrf2 [31]. In accordance with the Keap1-dependent Nrf2 activation pathway, CAPE binding to Keap1 by interacting with cysteine residues disrupts the Keap1-Nrf2 complex allowing Nrf2 to translocate to the nucleus and finally causes the induction of ARE promoter activity and HO-1 expression. It was also reported that oxidizable diphenols such as CAPE that carry a catechol moiety up-regulate ARE-related genes by inducing the Keap1-dependent Nrf2 activation signaling pathway [3]. In addition, the blocking of ERK by U126 treatment did not totally alleviate the induction of ARE promoter activity enhanced by CAPE treatment. Therefore, these results suggest that CAPE may induce Nrf2 activation without the ERK signaling pathway to result in the expression of genes containing ARE copy.

### 2.4. CAPE Partially Attenuates Cellular Oxidative Stress Induced by AAPH (2,2'-Azobis (2-amidinopropane) dihydrochloride) or H_2_O_2_ through Inducing HO-1 Expression 

**Figure 6:**
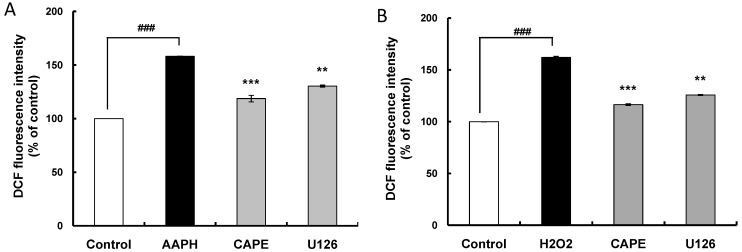
Cellular antioxidant capacity of CAPE against oxidative stress induced by AAPH (2,2'-azobis (2-amidinopropane) dihydrochloride) or H_2_O_2_ requires HO-1 induction through ERK activation (**A**,**B**). After HepG2 cells were first cultured in 96-well plates (5 × 10^5^/mL) with Dulbecco’s modified Eagle’s medium (DMEM) for 24 h, they were sequentially pre-incubated with 10 μM U126 for 30 min and 20 μM CAPE for 30 min and exposed to 60 μM AAPH or 1 mM H_2_O_2_ for 30 min. After HepG2 cells were then treated with 40 µM DCFH-DA (2',7'-dichlorodihyrofluorescein-diacetate) for 30 min in the dark, the cells were washed with Hank’s balanced salt solution (HBSS) and 2',7'-dichlorofluorescein (DCF), fluorescence intensity was measured at an excitation wavelength of 485 nm and an emission wavelength of 535 nm using a Tecan GENios fluorometric plate reader (Salzburg, Austria). Data are expressed as percentages of the value of untreated cells (mean ± standard deviation, *n* = 3), ^###^ *p* <0.001* vs.* control, *** *p* <0.001* vs.* AAPH, ** *p* <0.01* vs.* AAPH.

Cellular antioxidant capacity of antioxidants results from direct and indirect antioxidant capacity. Direct antioxidant capacity is defined as the capacity of the antioxidant itself to scavenge reactive oxygen and nitrogen species by donating hydrogen or electrons. Indirect antioxidant capacity is the capacity of an antioxidant to provide defense against oxidative stress through inducing the expression of ARE-related phase II detoxifying and antioxidant genes. To determine the contributing effect of CAPE through inducing HO-1 expression on its indirect antioxidant capacity, the cellular antioxidant capacity of CAPE was investigated using 2',7'-dichlorodihyrofluorescein-diacetate (DCFH-DA) in the presence of the oxidative inducers 2,2'-azobis (2-amidinopropane) dihydrochloride (AAPH) and H_2_O_2_ with U126 treatment. As cell viability over 95% was observed with 60 μM AAPH, 1 mM H_2_O_2_, and 20 μM CAPE (data not shown), HepG2 cells were pre-incubated with 20 μM CAPE for 30 min and exposed to 60 μM AAPH or 1 mM H_2_O_2_ for 30 min. The cells were then treated with DCFH-DA, which is a fluorescent probe that detects ROS (reactive oxygen species), for 30 min to measure intracellular oxidative stress induced by AAPH or H_2_O_2_. Intracellular oxidative stress in the HepG2 cells treated with AAPH and H_2_O_2_ increased by 158.0% and 162.0% respectively compared with the control group (Figure 6A,B); however, CAPE treatment, as expected, ameliorated the AAPH- and H_2_O_2_-induced oxidative stress to 119.1% and 116.2% respectively. This cellular antioxidant capacity of CAPE observed in HepG2 cells may be due to both direct and indirect antioxidant capacity as antioxidant activity exerted by CAPE both* in vitro* and* in vivo* was reported [11,13]. The cellular antioxidant capacity was determined at 1.5 h after treatment with CAPE in the cellular antioxidant capacity assay system applied in this study. In a short period, CAPE can scavenge peroxyl radicals from AAPH and H_2_O_2_ because it may donate hydrogen from the hydroxyl groups in the catechol moiety. Meanwhile, CAPE induced Nrf2 accumulation in the nucleus in 1 h (Figure 3A,B) and ARE promoter activity in 3 h (Figure 1B), indicating that CAPE can exert indirect antioxidant capacity by inducing phase II detoxifying and antioxidant enzymes such as HO-1. The indirect antioxidant capacity of CAPE that occurs by Keap1-dependent Nrf2 activation cannot be ignored in case CAPE is oxidized to its corresponding quinone. 

**Figure 7:**
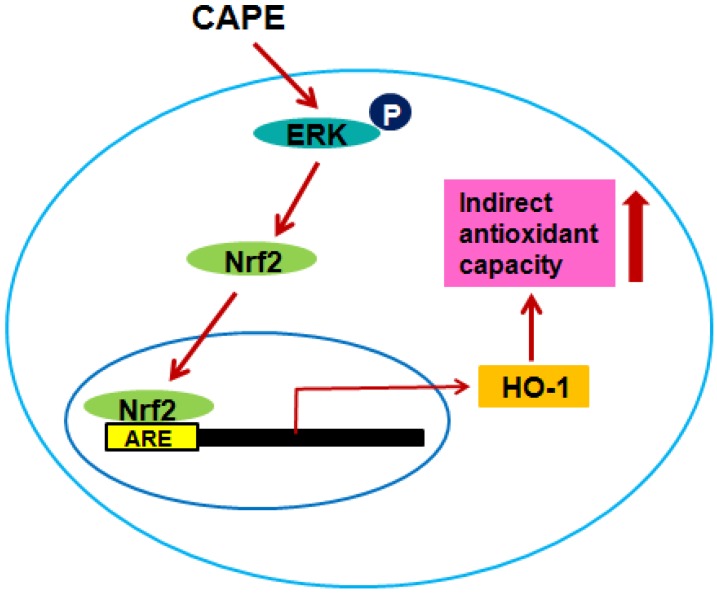
The ERK-Nrf2 signaling pathway of CAPE contributing to indirect cellular antioxidant capacity.

To clarify whether the ERK signaling pathway may be involved in the indirect antioxidant capacity of CAPE, HepG2 cells were pretreated with 10 μM U126 as an ERK inhibitor for 30 min before CAPE treatment. Although treatment with the ERK inhibitor U126 significantly (*p* < 0.01) ameliorated the cellular antioxidant capacity observed in CAPE treatment against oxidative stress significantly (*p* < 0.001) induced by AAPH or H_2_O_2_, it did not completely inhibit the cellular antioxidant capacity displayed by CAPE (Figure 6A,B). A possible explanation for this observation is that some other indirect antioxidant capacity not coupled with the ERK signaling pathway and direct antioxidant capacity may account for a certain portion of the cellular antioxidant capacity of CAPE. In line with the proposal that there is some other indirect antioxidant capacity excluding the ERK signaling pathway, both Nrf2 accumulation in the nucleus and ARE promoter activity induced by CAPE were only partly suppressed by ERK inhibitor U126 (Figure 5C,E respectively). Accordingly, the overall results provide solid evidence that cellular antioxidant capacity exerted by CAPE in HepG2 cells may be partially attributed to induction of HO-1 expression through the ERK-Nrf2 signaling pathway as depicted in Figure 7; however, further investigation is required to elucidate precisely the molecular signaling mechanisms involved in the indirect antioxidant capacity of CAPE in HepG2 cells.

## 3. Experimental Section 

### 3.1. Reagents

CAPE, AAPH, neocuproine, DMEM (Dulbecco’s modified Eagle’s medium), fetal bovine serum (FBS), 3-(4,5-dimethylthiazol-2-yl)-2,5-diphenyltetrazolium bromide (MTT), Triton X-100, HBSS, DCFH-DA, phosphate buffered saline (PBS, pH 7.4), SF, U126, and dimethylsulfoxide (DMSO) were purchased from Sigma Aldrich (St. Louis, MO, USA). FuGENE HD reagent (Promega, Madison, WI, USA), Dual-Glo luciferase assay kit, pGL4.37 (*luc2P*/ARE/Hygro) vector, and pGL4.74 (*hRluc*/TK) vector were purchased from Promega (Madison, WI, USA). CAPE was purchased from Cayman Chemical Company (Ann Arbor, MI, USA). Maxime PCR PreMix Kit and AccuPowerCycleScript RT PreMix were purchased from iNtRON biotechnology and Bioneer (Seoul, Korea). Antibodies purchased from Santa Cruz Biotechnology (Santa Cruz, CA, USA) included anti-SOD1, anti-HO-1, anti-catalase (CAT), anti-NAD(P)H:quinone oxidoreductase 1 (NQO1), anti-glutamyl systeine synthase (GCS), anti-Nrf2, anti-phospho-JNK, anti-phospho-PKC, and anti-phospho-Akt (Ser473). Anti-p44/42MAPK (ERK1/2), anti-phospho-p44/42MAPK (ERK1/2), and anti-glutathione S-transferase (GST) were purchased from Cell Signaling Technology (Beverly, MA, USA). HepG2 cells were obtained from the Korea Cell Line Bank (KCLB No. 88065, Seoul, Korea). 

### 3.2. Transient Transfection and Antioxidant Response Element (ARE)-Luciferase Assay

HepG2 cells were first cultured in 96-well plates (5 × 10^5^/mL) with DMEM for 24 h. This assay was performed using FuGENE HD reagent. After the HepG2 cells were transiently transfected with different plasmids, the cells were incubated with different concentrations of test samples dissolved in 10% DMSO for 24 h. ARE-luciferase activities were measured by the Dual-Glo Luciferase Assay System in accordance with the manufacturer’s protocol (Promega, Madison, WI, USA). Multiwell plate containing HepG2 cells was removed from the incubator. For measuring ARE luciferase activity, a volume of Dual-Glo Luciferase reagent equal to the culture medium volume was added to each well and mixed. After 10 min, a volume of Dual-Glo Stop & Glo reagent equal to the original culture medium volume was added to each well and mixed to determine Renilla luciferase activity. Luminescence was measured using a Tecan GENios fluorometric plate reader. The ratio of luminescence from ARE luciferase activity to that from Renilla luciferase activity was calculated.

### 3.3. Reverse Transcription-Polymerase Chain Reaction (RT-PCR)

Total RNA was extracted from HepG2 cells using a Trizol reagent (Invitrogen, Carlsbad, CA, USA) in accordance with the manufacturer’s instructions. From 1 μg of total RNA, cDNA was generated using the AccuPower CycleScript RT PreMix (Bioneer, Seoul, Korea). PCR reactions were performed with the following couples of primers: 5'-GTGTAAGGACCCATCGGAGA-3' and 5'-GTGTAAGGACCCATCGGAGA-3' for HO-1; 5'-GGTGTGGCCGATGTGTCTAT-3' and 5'-GGGCGATCCCAATTACACCA-3' for SOD-1; 5'-ATGCAGGACAATCAGGGTGG-3' and 5'-CCGCACAAAGGTGTGAATCG-3' for CAT; 5'-CAGCGCCCCGGACTGCACCAGAGCC-3' and 5'-GGGAAGCCTGGAAAGATACCCAGA-3' for NQO-1; 5'-GGAAGGTGTGTTTCCTGGACT-3' and 5'-TATTATACACACGGGCTGAGAGG-3' for GCS; and 5'-TCCATCCGGTGGCTCCTGGC-3' and 5'-GGGCAGAAGAAGGATCATTT-3' for GST. Annealing temperatures were programmed at 60 °C except for HO-1 and Nrf2 (58 °C). The PCR products were analyzed using 1.5% agarose gel electrophoresis, stained with EtBr (ethidium bromide), and visualized by UV detector.

### 3.4. Western Blot Analysis

HepG2 cells were grown in 6-well plates for 24 h and then incubated with CAPE in the presence or absence of U126 at 37 °C for different time periods. HepG2 cells were lysed in a radio immuno precipitation assay (RIPA) buffer (50 mM Tris-HCl (pH 8.0), 1% NP-40, 0.5% sodium deoxycholate, 150 mM NaCl, and 1 mM phenylmethanesulfonyl fluoride (PMSF)) that contained a phosphatase inhibitor cocktail. For nuclear protein extraction, after the harvested cells were rinsed twice with cold PBS, they were scraped and centrifuged at 15,800× *g* for 2 min. The pellet was then resuspended in 400 μL of buffer A (10 mM hydroxyethyl piperazineethanesulfonic acid (HEPES) (pH 7.9), 10 mM KCl, 2 mM MgCl_2_, 1 mM dithiothreithol (DTT), 0.5 mM ethylenediamine tetra-acetic acid (EDTA) (pH 7.9), 0.1 μM PMSF, and 1× protease inhibitor cocktail). After 15 min on ice, the lysate was centrifuged at 15,800× *g* for 2 min and the supernatant (cytosolic extract) was stored at 4 °C. The nuclear pellet was resuspended in buffer B (50 mM HEPES (pH 7.9), 50 mM KCl, 0.3 mM NaCl, 1 mM DTT, 1 mM EDTA (pH 7.9), 0.1 μM PMSF, and 10% glycerol) for 10 min at 4 °C. The resuspended pellet was centrifuged at 18,000× *g* for 10 min at 4 °C and the supernatant (nuclear extract) was stored at −70 °C. The lysed cells and extracted nuclear and cytosol proteins were subjected to electrophoresis using sodium dodecylsulfate-polyacrylamide gel electrophoresis (SDS-PAGE) and transferred to nitrocellulose membranes. The membranes were reacted with primary antibodies for 12 h and then incubated with the appropriate horseradish peroxide-conjugated secondary antibodies for 1 h at room temperature. The proteins on the membranes were detected with an EZ-western Lumi Pico detection kit (DoGEN, Seoul, Korea) and visualized using an LAS4000 chemiluminescent image analyzer (Fuji, Tokyo, Japan).

### 3.5. Observation of Fluorescence Imaging for Nrf2 and Nucleus

Observations were performed by confocal imaging as described by Grindel* et al.* [32]. Following treatment, HepG2 cells were rinsed twice with PBS and incubated for 15 min at room temperature with 3.7% paraformaldehyde in PBS. The cells were washed again with PBS followed by 10 min incubation at room temperature in 0.1% Triton X-100 in PBS. After washing with PBS, the cells were blocked in 5% skimmed milk in PBS for 45 min at room temperature. The blocking solution was removed and replaced with 1:500 Nrf2 primary antibody in 5% skimmed milk, and the cells were left overnight at 4 °C. After overnight incubation, the cells were washed for 10 min three times in PBS. The washing solution was removed and replaced with 1:2500 AlexaFlour 546 conjugated goat anti-rabbit secondary antibody (Invitrogen, Carlsbad, CA, USA) in PBS, and the cells were incubated for 45 min at room temperature in the dark. Then the cells were washed for 10 min three times in PBS. Images were acquired with an LSM5 live configuration Variotwo VRGB microscope (Zeiss, Jena, Germany) equipped with an oil immersion lens. Fluorescence imaging was achieved using a laser for 405 nm (blue, death-associated protein 1 (DAPI) nuclear staining) and 535 nm (red, anti-Nrf2) excitation.

### 3.6. Cell Viability Assay

The tetrazolium dye colorimetric test (MTT) was used to determine the viability of HepG2 cells. The MTT assay is based on the ability of functional mitochondria to catalyze the reduction of 3-(4,5-dimethylthiazol)-2,5-diphenyltetrazolium bromide to insoluble purple formazan, the concentration of which can be measured spectrophotometrically. HepG2 cells were first cultured in 96-well plates (5 × 10^5^ cells/well) for 24 h, washed twice using PBS, and pretreated with different concentrations of test samples of AAPH, H_2_O_2_, and CAPE. After 24 h incubation, MTT reagent was added to each well and the plate was incubated at 37 °C for 2 h. The media was removed and the plate was washed twice with PBS (pH 7.4). The intracellular insoluble formazan was dissolved in DMSO. The absorbance of each cell was then measured at 570 nm using an ELISA reader (Tecan, Salzburg, Austria) and the percentage viability was calculated.

### 3.7. Cellular Antioxidant Capacity

Cellular oxidative stress due to reactive oxygen species (ROS) generated by AAPH or H_2_O_2_ was measured spectrofluorometrically using the DCFH-DA method [33]. DCFH-DA diffuses through the cell membrane and is hydrolyzed enzymatically by intracellular esterase to non-fluorescent 2',7'-dichlorodihydrofluorescein (DCFH), which is rapidly oxidized to highly fluorescent 2',7'-dichlorofluorescein (DCF) in the presence of ROS. HepG2 cells were first cultured in 96-well plates (5 × 10^5^/mL) with DMEM for 24 h. After the cells were pre-incubated with 10 μM U126 for 30 min, they were incubated in different concentrations of CAPE dissolved in DMSO for 30 min. Then, the medium was discarded and the wells were gently washed twice with PBS. HBSS, which is fluorescently stable, was then added to each well instead of normal medium, and AAPH or H_2_O_2_ was used as an oxidative stress inducer. After the cells were treated with 60 μM AAPH or 1 mM H_2_O_2_ for 30 min, DCFH-DA was added to the culture plates at a final concentration of 40 μM and the cells incubated for 30 min at 37 °C in the dark. After incubation, the cells were washed with HBSS, and the DCF fluorescence intensity was measured at an excitation wavelength of 485 nm and an emission wavelength of 535 nm using a Tecan GENios fluorometric plate reader.

### 3.8. Statistical Analysis

All data are presented as mean ± standard deviation. Statistical analysis was carried out using the SPSS statistical package (SPSS, Chicago, IL, USA) program, and the significance of each group was verified with one-way analysis of variance (ANOVA) followed by Duncan’s test or Student’s *t*-test. A *p* value <0.05 was considered significant.

## 4. Conclusions 

From our results it is proposed that an indirect antioxidant activity of CAPE against oxidative stress in hepatic HepG2 cells is in part attributable to induction of HO-1, which is regulated by Keap1-independent Nrf2 activation relying on post-translational phosphorylation of ERK. This may well have implications in the use of propolis carrying CAPE for the amelioration of oxidative stress-related diseases such as atherosclerosis, hypertension, and neurodegeneration.

## References

[1] Dinkova-Kostova A.T., Talalay P. (2008). Direct and indirect antioxidant properties of inducers of cytoprotective proteins. Mol. Nutr. Food Res..

[2] Dinkova-Kostova A.T., Cheah J., Samouilov A., Zweiser J.L., Bozak R.E., Hicks R.J., Talalay P. (2007). Phenolic Michael reaction acceptors: combined direct and indirect antioxidant defense against electrophiles and oxidants. Med. Chem..

[3] Dinkova-Kostova A.T., Wang X.J. (2011). Induction of the Keap1/Nrf2/ARE pathway by oxidizable diphenol. Chem. Biol. Interact..

[4] Bryan H.K., Olayanju A., Goldring C.E., Park B.K. (2013). The Nrf2 cell defence pathway: Keap1-dependent and -independent mechanisms of regulation. Biochem. Pharmacol..

[5] Li Y., Paonessa J.D., Zhang Y. (2012). Mechanism of chemical activation of Nrf2. PLoS One.

[6] Park J.H., Lee J.K., Kim H.S., Chung S.T., Eom J.H., Kim K.A., Chung S.J., Paik S.Y., Oh H.Y. (2004). Immunomodulatory effect of caffeic acid phenethyl ester in Balb/c mice. Int. Immunopharmacol..

[7] Baird L., Dinkova-Kostova A.T. (2011). The cytoprotective role of the Keap1-Nrf2 pathway. Arch. Toxicol..

[8] Itoh K., Tong K.I., Yamamoto M. (2004). Molecular mechanism activation of Nrf2-Keap1 pathway in regulation of adaptive response to electrophiles. Free Radic. Biol. Med..

[9] Son S., Lewis B.A. (2002). Free radical scavenging and antioxidant activity of caffeic acid amide and ester analogues: Structure-activity relationship. J. Agric. Food Chem..

[10] Russo A., Longo R., Vanella A. (2002). Antioxidant activity: Role of caffeic acid phenethyl ester and galangian. Fitoterapia.

[11] Ahn M.R., Kunimass K., Kumazawa S., Nakayama T., Kaji K., Uto Y., Hori H., Nagasawa H., Ohta T. (2009). Correlation between antiangiogenic activity and antioxidant activity of various components from propolis. Mol. Nutr. Food Res..

[12] Wang X., Stavchansky S., Bowman P.D., Kerwin S.M. (2006). Cytoprotective effect of caffeic acid phenethyl ester (CAPE) and catechol ring-fluorinated CAPE derivatives against menadione-induced oxidative stress in human endothelial cells. Bioorg. Med. Chem..

[13] Yilmaz H.R., Uz E., Yucel N., Altuntas I., Ozcelik N. (2004). Protective effect of caffeic acid phenethyl ester (CAPE) on lipid peroxidation and antioxidant enzymes in diabetic rat liver. J. Biochem. Mol. Toxicol..

[14] Okutan H., Ozcelik N., Yilmaz H.R., Uz E. (2005). Effects of caffeic acid phenethyl ester on lipid peroxidation and antioxidant enzymes in diabetic rat heart. Clin. Biochem..

[15] Mapesa J.O., Waldschmit N., Schmoeller I., Blume C., Hofmann T., Mahungu S., Clavel T., Haller D. (2011). Catechols in caffeic acid phenethyl ester are essential for inhibition on TNF-mediated IP-10 expression through NF-κB-dependent but Ho-1- and p38-independent mechanisms in mouse intestinal epithelial cells. Mol. Nutr. Food Res..

[16] Borrelli F., Maffia P., Pinto L., Ianaro A., Russo A., Capasso F., Lalenti A. (2002). Phytochemical compounds involved in the anti-inflammatory effects propolis extract. Fitoterapia.

[17] Ho C.C., Chou M.Y., Chen F.L., Hu C.C., Chen C.S., Lu G.Y., Yang C.C. (2005). Effects of CAPE-like compounds on HIV replication *in vitro* and modulation of cytokines *in vivo*. J. Antimicrob. Chemother..

[18] Chung T.W., Moon S.K., Chang Y.C., Ko J.H., Lee Y.C., Cho G., Kim S.H., Kim J.G., Kim C.H. (2004). Novel and therapeutic effect of caffeic acid phenethyl ester on hepatocarcinoma cells: Complete regression of hepatoma growth and metastasis by dual mechanism. FASEB J..

[19] Balogun E., Hoque M., Gong P., Killeen E., Green C.J., Foresti R., Alam J., Motterlini R. (2003). Curcumin activates the haem oxygenase-1 gene via regulation of Nrf2 and the antioxidant-responsive element. Biochem. J..

[20] Alía M., Ramos S., Mateos R., Bravo L., Goya L. (2005). Response of the antioxidant defense system to *ter*-buty hydroperoxide and hydrogen peroxide in a human hepatoma cell line (HepG2). J. Biochem. Mol. Toxicol..

[21] Goya L., Mateos T., Bravo L. (2007). Effect of the olice oil phenol hydroxytyrosol on human hepatoma HepG2 cells. Eur. J. Nutr..

[22] Wang X., Stavchansky S., Zhao B., Byum J.A., Kerwin S., Bowman P.D. (2008). Cytoprotection of human endothelial cells from menadione cytotoxicity by caffeci acid phenethyl ester: The role of heme oxygenase-1. Eur. J. Pharmacol..

[23] Wang X., Stavchansky S., Kerwin S.M., Bowman P.D. (2010). Structure-activity relationships in the cytoprotective effect of caffeic acid phenethyl ester (CAPE) and fluorinated derivatives: Effects on heme oxygenae-1 induction and antioxidant activities. Eur. J. Pharmacol..

[24] Scapagnini G., Foresti R., Calabrese V., Gluffrida Stella A.M., Green C.J., Motterlini R. (2002). Caffeic acid phenethyl ester and curcumin: A novel class of heme oxygenase-1 inducers. Mol. Pharmacol..

[25] Scapagnini G., Vasto S., Abraham N.G., Caruso C., Zella D., Fabio G. (2011). Modulation of Nrf2/ARE pathway by food polyphenols: a nutritional neuroprotective stragety for cognitive and neurodegenerative disorders. Mol. Neurobiol..

[26] Suzuki K., Tanaka I., Nakahishi I., Kurematsu A., Yakumaru H., Ikota N., Ishihara H. (2006). Drastic effect of several caffeic acid derivatives on the induction of heme oxygenase-1 expression revealed by quantitative real-time RT-PCR. Biofactors.

[27] Yasui N., Nishiyama E., Juman S., Negishi H., Miki T., Yamori Y., Ikeda K. (2013). Caffeic acid phenethyl ester suppresses oxidative stress in 3T3-L1 adipocytes. J. Asian Nat. Prod. Res..

[28] Liu R., Chen H., Bai H., Zhang W., Qin X., Zhang X., Li W., Liang X., Hai C. (2013). Suppression of nuclear factor erythroid 2-related factor 2 via extracellular signal-regulated kinase contributes to bleomycin-induced oxidative stress and fibrogenesis. Toxicol. Lett..

[29] Gu J., Sun X.C., Wang G.N., Li M.M., Chi M. (2011). Icariside II enhances Nrf2 nuclear translocation to upregulate phase II detoxifying enzyme expression coupled with the ERK, Akt and JNk signaling pathways. Molecules.

[30] Yuan X.L., Xu C.J., Pan Z., Keum Y.S., Kim J.W., Shen G.X., Yu S.W., Oo K.T., Ma J.J., Kong A.T. (2006). Butylated hydroxyanisole regulates ARE-mediated gene expession via Nrf2 coupled with ERK and JNK signaling pathway in HepG2 cells. Mol. Carcinog..

[31] Kim H.J., Kim W.S., Yum S.W., Hong S.C., Oh J.E., Lee J.W., Kwak M.K., Park E.J., Na D.H., Jung Y.J. (2013). Caffeic acid phenethyl ester activation of Nrf2 pathway is enhanced under oxidative state: Structural analysis and potential as a pathologically targeted therapeutic agent in treatment of colonic inflammation. Free Radic. Biol. Med..

[32] Grindel B.J., Rohe B., Safford S.E., Bennett J.J., Farach-Carson M.C. (2011). Tumor necrosis factor-α treatment of HepG2 cells mobilizes a cytoplasmic pool of Erp57/1,25D3-MARRS to the nucleus. J. Cell. Biochem..

[33] Lautraite S., Bigot-Lasserre D., Bars R., Carmichael N. (2003). Optimization of cell-based assays for medium through screening of oxidative stress. Toxicol. In Vitro.

